# Maternal satisfaction and birth experiences after elective induction vs. spontaneous onset in late-term pregnancy: a register-based study

**DOI:** 10.1186/s12884-025-07818-3

**Published:** 2025-06-19

**Authors:** Sahruh Turkmen, Linnea Binfare

**Affiliations:** 1 Department of Clinical Sciences, Obstetrics and Gynecology, Sundsvalls Research Unit, Umeå University, Umeå, 90185 SE Sweden; 2 Department of Obstetrics and Gynecology, Sundsvall County Hospital, Sundsvall, 85186 SE Sweden

**Keywords:** Prolonged pregnancy, Induction of labour, Spontaneous onset of labour, Obstetric outcome, Satisfaction with birth, Childbirth experience

## Abstract

**Background:**

Earlier studies highlight that a positive birth experience enhances both short-term recovery and long-term maternal well-being. However, the factors influencing this experience are complex and not yet fully understood. We aimed to investigate the influence of labour commencement method on late-term pregnant women’s satisfaction with care and the birth experience, and to determine whether women’s perception of their birth experience changes over time.

**Methods:**

In this register-based retrospective cohort study, we included pregnant women in late term (≥ 41 + 0 to < 42 + 0) who gave birth in Sweden during 2020–2021. Eligible women were classified into two groups: spontaneous onset of labour (SOL) and induced onset of labour (IOL). Women’s satisfaction with care at birth and the childbirth experience at 8 weeks and 1 year postpartum were measured with a visual analogue scale, where 0 indicates “very unsatisfied” and 10 “very satisfied”.

**Results:**

Satisfaction with care at the time of discharge from the hospital was significantly different between the IOL and SOL groups, with mean scores of 6.53 ± 3.34 and 6.97 ± 3.34, respectively (*P* = 0.007). Furthermore, the IOL group reported a less positive birth experience at 8 weeks (7.15 ± 2.37 and 7.74 ± 2.17, respectively, *P* = 0.004) and 1 year postpartum (6.87 ± 2.40 and 7.53 ± 2.15, respectively, *P* = 0.002) compared to the SOL group. Both groups experienced a decline in birth experience positivity from 8 weeks to 1 year postpartum (*P* < 0.001 in both groups). Common factors influencing satisfaction with care and birth experience included parity, heavy bleeding during labour, and the mode of delivery.

**Conclusion:**

Satisfaction with care during labour and women’s childbirth experiences differed between the SOL and IOL groups, indicating a relationship between labour commencement method and satisfaction scores. Women in the SOL group were more satisfied with care at birth and reported a more positive birth experience at both 8 weeks and 1 year postpartum compared to the IOL group. Over time, women’s childbirth experience scores in both groups may change, becoming less positive 1 year after birth compared to 8 weeks postpartum.

**Trial registration:**

Retrospectively registered.

## Background

In high-income countries, where maternal and neonatal mortality and morbidity rates are low, there is a growing focus on the satisfaction with care and the maternal childbirth experience in maternity care. It is estimated that between 4.3% and 34% of women experience negative childbirth outcomes [1–3]. Studies have also shown that the birth experience can have short- and long-term consequences for a woman’s health and an indirect effect on her family [4]. A poor labour experience may affect a woman’s future pregnancies and reproduction, because of fear of becoming pregnant again after a previous negative birth experience [5, 6]. The degree of satisfaction with the care received and the experience of giving birth in the case of induced labour is not fully understood [7, 8].

The proportion of induced births varies between maternity units in different countries [9, 10]. Recently, The National Board of Health and Welfare in Sweden has emphasized that healthcare providers should conduct an assessment and plan for labour induction at the start of gestational week 41. The objective is to ensure that all pregnant individuals are either in labour or have given birth by the beginning of week 42 [11]. This approach aims to balance the benefits of reducing perinatal mortality with the risks associated with prolonged pregnancies. Consequently, the proportion of induced births in Sweden has increased from 8% in 1993 to 25% in 2020 [12]. However, patients’ childbirth experiences and satisfaction with birth are variable and involve various underlying mechanisms that are influenced by many factors [13]. Some studies suggest that women undergoing induction of labour often report a poorer childbirth experience compared to those with spontaneous onset of labour. The main contributing factors identified include increased pain, prolonged labor, unplanned cesarean sections, and a lack of support from caregivers [1]. However, other studies indicate that labour induction does not always result in a negative childbirth experience and may even have positive outcomes in certain cases [14]. Additionally, a Swedish study (SWEPIS) comparing induction at 41 weeks versus expectant management until 42 weeks found no significant differences in overall childbirth experience between the two groups [15]. It remains unclear how the method of labour commencement affects women’s satisfaction with care during birth and their childbirth experiences after delivery [16].

A woman’s satisfaction with childbirth care and her overall healthcare experience is predominantly measured using questionnaires, with the visual analogue scale (VAS) being one of the most widely utilized methods [17, 18]. However, findings from some studies raise concerns about the interpretation of VAS results. One study suggests that VAS primarily reflects perceptions of safety and a woman’s participation in labor but does not sufficiently account for the professional support received [19]. This limitation underscores the need for caution when analyzing research findings and developing appropriate follow-up measures for individuals with negative birth experiences. Despite these concerns, VAS remains a valuable tool for mood assessment, demonstrating high compliance and reliability when participants receive clear guidance [20].

The aim of this study was to: (a) investigate the associations between the method of labour commencement and the satisfaction with care and birth experience of low-risk pregnant women in late term (≥ 41 weeks + 0 days to ≤ 41 + 6.days) [21], (b) evaluate the influence of maternal characteristics and maternal–neonatal outcomes on women’s satisfaction with care and birth experience, and (c) examine whether a woman’s perception of her birth experience changes over time, specifically comparing the time points of 8 weeks and 1 year after delivery.

## Methods

We performed a retrospective nationwide cohort study of women who gave birth between gestational week ≥ 41 + 0 and week < 42 + 0 (late-term) between 1 January 2020 and 31 December 2021. The Ethics Review Authority of Stockholm Division 1 Medicine approved the study (Dnr 2020–06274).

The maternal -foetal data were extracted from the Swedish Pregnancy Register (Svenska graviditetsregistret), which includes data for > 90% of all pregnancies in Sweden. The register encompasses information from the first antenatal visit through to the postpartum follow-up. It aims to improve the quality of care and support research on maternal and neonatal health. Woman’s satisfaction with care at birth and their experience of healthcare were assessed using the web-based questionnaire ‘Graviditetsenkäten’ developed by the national organization Sveriges Kommuner och Regioner (SKR). The questionnaire data is available in the Pregnancy Register.

Women with late-term, low-risk pregnancies were divided in two groups: those who gave birth following elective induction of labour due to being in late-term (the IOL group) and those who gave birth after expectant management and spontaneous onset of labour (the SOL group) in the same gestational week.

### Inclusion and exclusion criteria

Pregnant women were included if they were 18 years or over, had a spontaneous pregnancy with one foetus in cephalic presentation at a gestational age of between ≥ 41 weeks + 0 days and < 42 weeks + 0 days, based on an ultrasound examination in the early second trimester. Women were excluded if they had more than one earlier caesarean section or other uterine surgery, missing data on VAS scores at one of the three assessment points (at birth, 8 weeks, and 1 year after birth), any contraindications for vaginal birth, diabetes mellitus type 1 or 2, gestational diabetes treated with medication, hypertensive gestational disorder, a history of or on-going psychological disorders, observed oligohydramniosis (deepest vertical pocket of amnion fluid < 20 mm on ultrasound), or a small-for-gestational-age foetus (SGA is defined as 2 standard deviations below the standard growth curve) [22] including intra-uterine growth retardation (IUGR), perinatal mortality (defined as stillbirth after gestational week 22 and in newborns before days 0–7 after delivery), diagnosed foetal abnormalities, and other maternal diseases that can affect the progress of pregnancy until 42 weeks of gestation.

### Study parameters

The women’s pooled demographic parameters were age, body mass index (BMI), parity, tobacco use at the time of registration in maternity care, previous caesarean delivery, fear of childbirth, education level (i.e., with or without a school diploma), country of birth (immigration status), occupation, co-habitation with partner, and number of visits to the maternity-care centre during pregnancy.

The maternal outcomes were the woman’s satisfaction with care provided during birth at the time of discharge from hospital and her perceived experience of the birth at 8 weeks and 1 year after delivery, the mode of delivery (caesarean section, forceps, vacuum-assisted delivery, or normal vaginal delivery), duration of labour (hours, from the beginning of the active phase to delivery), third and fourth degrees of perineal tear, episiotomy, epidural anaesthesia (EDA), oxytocin use in labour, and postpartum haemorrhage > 1000 ml.

The neonatal outcomes were birth weight, newborn’s sex [23], Apgar score < 7 at 5 min, and potential hydrogen (pH) of the umbilical artery blood at birth.

To support women who have been unsatisfied with healthcare at birth, all the delivered women in Sweden are routinely asked about their satisfaction with birth. The patient is asked to complete a VAS at the time of discharge from hospital. The VAS varies from 0 (very unsatisfied) to 10 (very satisfied). The specific question that is answered using the VAS is “Are you satisfied with the care provided during your delivery?”.

To add more qualitative information concerning the delivery and the women’s personal feelings and interpretations of the birth process (the childbirth experience), we retrieved information from the web-based questionnaire “Graviditetsenkäten”, which was developed by the national organization Sveriges Kommuner och Regioner (SKR) [24]. All women who have undergone childbirth in Sweden are offered the opportunity to fill in the questionnaire three times: in gestational week 25, and 8 weeks and 1 year after delivery [25]. We included and processed the women’s answers at the postpartum time points (8 weeks and 1 year after delivery) using only one specific objective question (“How did you experience this birth?”), which was asked on both occasions. The women indicated their childbirth experiences by marking a cross along a line of exactly 10 cm in length (i.e., the VAS), with endpoints of “very poor experience = 0” to “very positive experience = 10”.

### Statistical analysis

All statistical analyses were performed using Statistical Package for Social Sciences (SPSS) version 29 (IBM, Armonk, NY, USA). The primary statistical analysis compared women in late-term pregnancy who had delivered a child after IOL (IOL group) or after SOL (SOL group). The maternal and neonatal outcomes were tested with Pearson’s χ^2^ test (for dichotomous variables) or Student’s *t* test (for continuous variables). A paired-sample *t* test was used to analyse the changes in childbirth experience between two time points (8 weeks and 1 year postpartum). To examine the relationship between the childbirth experience, satisfaction with birth, the method of commencement of labour, and foetal–maternal outcomes, a multiple linear regression analysis was conducted. Potential confounders were identified as age, parity, education, occupation, country of birth, cohabitation with partner, fear of childbirth, and previous caesarean delivery. The potential mediators were epidural anaesthesia, oxytocin use during labour, mode of delivery, episiotomy, duration of labour, perineal injury, bleeding > 1000 ml, Apgar score < 7 at 5 min, pH, and foetal birth weight. Adjustments for confounders and mediators were performed in two steps: In the first step, we adjusted for the potential confounders. Subsequently, we calculated the coefficients adjusted for the same confounders plus the mediators. The results are presented as the standardized beta-coefficient (β) and 95% confidence intervals (CI). The overall value of missing data (missing at random) was 4.7%. However, the highest percentages of missing data were observed for PH, and duration of labour (32%, 31%, and 26%, respectively). We conducted sensitivity analyses to address the missing data using multiple imputation with five repetitions. Statistical analyses were then performed on the pooled data set after imputation. A difference was statistically significant at *P* < 0.05.

## Results

**Fig. 1:**
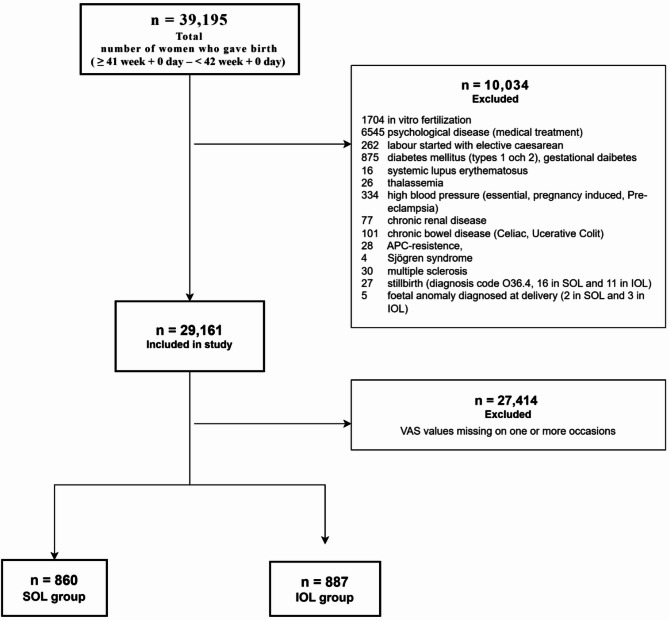
Flow chart of late-term pregnant patients included in the study. SOL: spontaneous onset of labour; IOL: induced onset of labour; n: number

The total number of pregnant women in late term who gave birth to one foetus with cephalic presentation after either SOL or IOL during study period was 39,195. Patients with more than one caesarean section deliveries were already excluded, and after applying additional exclusion criteria, the number of study patients was 29,161. Among these patients, 16,173 women after SOL and 12,988 after IOL. Women who did not answer the question about their childbirth experience 8 weeks (73%) and/or 1 year after delivery (72%) were also excluded. The group that responded to the questionnaires across all three occasions comprised a significantly higher proportion of women who were born in Sweden (81%), had a normal vaginal delivery both this time (81%) and in previous births (92%), were first-time mothers (56%), were employed (81%), held a university degree or higher education (60%), did not use tobacco (91%), and had a baby with a normal Apgar score at 5 min (99%). Consequently, the final number of patients who answered the question on all three occasions was *n* = 860 in the SOL group and *n* = 887 in the IOL group. (see Fig. 1).

The demographic data for the women in the SOL and IOL groups were compared. There was no significant difference in the ages of women who gave birth after induction of labour (IOL) compared to those who delivered after spontaneous onset of labour (SOL), with average ages of 31.27 ± 4.28 years and 31.48 ± 3.96 years, respectively, *P* = 0.284). However, women who gave birth after IOL had a significantly higher BMI (25.65 ± 5.10 kg/m²) than those who delivered after SOL (24.58 ± 4.12 kg/m²) (*P* < 0.001). The proportion of nulliparous women was higher in the IOL group than in the SOL group (58.5% and 50.0%, respectively, *P* < 0.001), as was those who cohabited with a partner (95.6% and 97.2%, respectively, *P* = 0.047).

The proportions of women with one previous caesarean delivery, tobacco user, unemployed, with education, with a fear of childbirth, and those with female babies were not different between the IOL and SOL groups. The patient demographics are provided in Table 1.

**Table 1:** Women’s demographic characteristics according to study group

	SOL group (*n* = 860) Mean ± SD (*n*), or % (*n*)	IOL group (*n* = 887) Mean ± SD (*n*), or % (*n*)	*P* value
**Age (years)**	31.48 ± 3.96 (860)	31.27 ± 4.28 (887)	0.284
**BMI (kg/m**^**2**^)	24.58 ± 4.12 (840)	25.65 ± 5.10 (852)	< 0.001
- **Less than 18.49**	1.2 (10)	1.9 (16)	
- **18.5–24.9**	59.2 (497)	52.2 (445)	
- **25–29.9**	29.5 (248)	29.1 (248)	
- **≥ 30**	10.1 (85)	16.8 (143)	
**Parity**			
- **Nulliparous**	50.0 (430)	58.5 (519)	< 0.001
- **Multiparous**	50.0 (430)	41.5 (368)	
**Previous caesarean delivery**:			
- **Yes**	5.3 (46)	7.0 (62)	0.092
- **No**	94.7 (814)	93.0 (825)	
**Education**			
- **Primary school**	1 (9)	0.1 (1)	0.228
- **Up to and including secondary school**	22.3 (192)	24.9 (221)	
- **University or higher education**	67.3 (579)	62.7 (556)	
- **No/unknown**	9.4 (80)	12.3 (110)	
**Occupation**			
- **Unemployed**	6.2 (53)	7.3 (65)	0.191
- **Employed/Studying**	93.8 (807)	92.7 (822)	
**Cohabitation with partner**			
- **Yes**	97.2 (836)	95.6 (848)	0.047
- **No**	2.8 (24)	4.4 (39)	
**Tobacco user**			
- **Yes**	1.7 (15)	1.5 (13)	0.817
- **No**	98.2 (845)	97.5 (865)	
**Fear of childbirth**			
- **Yes**	4.1 (35)	5.4 (48)	0.114
- **No**	95.9 (825)	94.6 (839)	
**Number of maternity ward visits**	9.71 ± 2.46 (851)	10.03 ± 2.53 (876)	0.337
**Birth country (immigration)**			
- **Sweden**	86.7 (746)	89.4 (793)	0.050
- **Other countries**	13.3 (114)	10.6 (94)	
**Sex of newborn**			
- **Female**	49.0 (421)	45.1 (400)	0.059
- **Male**	51.0 (430)	54.9 (487)	

A comparison of the maternal obstetric outcomes showed that women who gave birth after IOL had a shorter, but not significantly different, duration of the active phase of labour (9.4 and 9.7 h, respectively, *P* = 0.217) and a higher rate of caesarean section compared to women who gave birth after SOL (10.9% and 4.3%, respectively, *P* < 0.001). Additionally, the IOL group had higher rates of episiotomy at birth (5.7% and 3.4%, respectively, *P* = 0.011), oxytocin use in labour (4.7% and 1.4%, respectively, *P* < 0.001), and epidural anaesthesia (15.5% and 8%, respectively, *P* = 0.005). However, the rate of women with perineal injury (grade 3 or 4), bleeding > 1000 ml at birth, did not differ between the groups (Table 2).

**Table 2:** Foetal and maternal outcomes

	SOL group (*n* = 860) Mean ± SD (*n*), or % (*n*)	IOL group (*n* = 887) Mean ± SD (*n*), or % (*n*)	*P* value
**Satisfaction with birth**	6.97 ± 3.34 (860)	6.53 ± 3.34 (887)	0.007
**Birth experience (8 weeks)**	7.74 ± 2.17 (860)	7.15 ± 2.37 (887)	0.004
**Birth experience (1 year)**	7.53 ± 2.15 (860)	6.87 ± 2.40 (887)	0.002
**Duration of Labour**	9.75 ± 5.59 (860)	9.43 ± 5.09 (887)	0.217
**Mode of delivery**			
- **Normal vaginal**	88.5 (761)	80.5 (714)	< 0.001
- **Vacuum assisted**	7.1 (61)	8.6 (76)	0.145
- **Forceps assisted**	0.1 (1)	0.0 (0)	0.492
**Perineal injury (3rd /4th degree)**			
- **Yes**	3.5 (30)	2.9 (26)	0.300
- **No**	96.5 (830)	97.1 (861)	
**Bleeding > 1 lit**			
- **Yes**	9.5 (82)	9.5 (93)	0.281
- **No**	10.5 (778)	89.5 (794)	
**Oxytocin use in labour**			
- **Yes**	1.4 (12)	4.7 (42)	< 0.001
- **No**	98.6 (848)	95.3 (845)	
**Perineotomy**			
- **Yes**	3.4 (29)	5.7 (51)	0.011
- **No**	96.6 (831)	94.3 (836)	
**Epidural anaesthesia**			
- **Yes**	8.0 (22)	15.5 (43)	0.005
- **No**	92.0 (253)	84.5 (235)	
**Apgar score (< 7 at 5 min)**			
- **Yes**	1.0 (9)	1.4 (12)	0.662
- **No**	99.0 (851)	98.6 (875)	
**Umbilical artery PH**	7.22 ± 0.08 (576)	7.21 ± 0.85 (621)	0.718
**Birth weight (g)**	3813 ± 433 (856)	3839 ± 477 (884)	0.280

Analysis of the foetal outcomes showed that the proportion of neonates with Apgar score < 7 at 5 min, the umbilical artery blood pH, and birth weight did not differ between the IOL and SOL groups (Table 2).

### Maternal birth experience over time: comparing satisfaction between IOL and SOL

In both the IOL and SOL groups, satisfaction scores with care at birth were lower compared with birth experience scores at 8 weeks (*P* < 0.001 for both groups) and 1 year postpartum (*P* < 0.001 for both groups). The women in the SOL group experienced a more positive childbirth experience and had a higher VAS score than women in the IOL group at both 8 weeks (7.74 ± 2.17 versus 7.15 ± 2.37, respectively, *P* = 0.004) and 1 year (7.53 ± 2.15 versus 6.87 ± 2.40, respectively, *P* = 0.002) after delivery (Table 2).

To evaluate how women’s perception of the childbirth experience changed during the postpartum period, we compared the VAS scores at 8 weeks and 1 year postpartum in both the SOL and IOL groups. In both groups, the VAS score decreased over time (SOL; 7.74 ± 2.17 and 7.53 ± 2.15, IOL; 7.15 ± 2.37 and 6.87 ± 2.40, at 8 weeks and 1 year, respectively, *P* < 0.001 in both groups).

### Women’s satisfaction with care at birth, evaluated at discharge from the maternity ward

Satisfaction with care at the time of discharge from the hospital was significantly different between the IOL and SOL groups, with mean scores of 6.53 ± 3.34 and 6.97 ± 3.34, respectively (*P* = 0.007).

To determine the associations between various factors and women’s satisfaction with care during birth, we used a multiple linear regression analysis with the predictors, including both the aforementioned confounders and mediators (Table 3).

**Table 3:** Associations between predictors and outcomes determined using a multiple linear regression analysis. β; standardized beta-coefficient

		Satisfaction with birth β, *p*-value			Childbirth experience at 8 weeks β, *p*-value			Childbirth experience at 1 year β, *p*-value		
		Unadjusted	Adjusted to Conf or Med	Adjusted to both Conf. Med	Unadjusted	Adjusted to each	Adjusted to both	Unadjusted	Adjusted to each	Adjusted to both
	Labour commencement method	**-0.64**,** 0.007**	0.021, 0.620	-0.014, 0.747	**-0.130**,** < 0.001**	**-0.141**,** < 0.001**	**-0.118**,** 0.003**	**-0.143**,** < 0.001**	**-0.146**,** < 0.001**	**-0.127**,** 0.002**
C	Age	0.009, 0.692	-0.007, 0.882	-0.009, 0.781	0.023, 0.346	0.024, 0.578	0.024, 0.564	0.007, 0.760	-0.032, 0.469	-0.029, 0.492
O	Parity	**0.141**,** < 0.001**	**0.225**,** < 0.001**	**0.187**,** < 0.001**	**0.217**,** < 0.001**	**0.278**,** < 0.001**	**0-198**,** < 0.001**	**0.224**,** < 0.001**	**0.240**,** < 0.001**	**0.154**,** < 0.001**
N	Education	0.013, 0.597	− 0.0.013, 0.813	-0.031, 0.592	0.014, 0.567	¨-0.020, 0.698	-0.055, 0.264	-0.011, 0.657	0.055, 0.292	0.017, 0.726
F	Occupation	0.006, 0.796	0.007, 0.900	0.002, 0.965	0.040, 0.098	-0.007, 0.898	0.019, 0.705	-0.013, 0.573	-0.091, 0.081	-0.071, 0.153
O	Country of birth	-0.016, 0.505	0.069, 0.102	0.063, 0.139	0.012, 0.620	0.059, 0.151	0.063, 0.110	-0.016, 0.505	0.013, 0.759	0.014, 0.721
U	Co-habitation with partner	**0.068**,** 0.005**	0.071, 0.094	0.073, 0.085	**0.068**,** 0.005**	0.064, 0.119	0.069, 0.079	**0.083**,** < 0.001**	0.033, 0.426	0.039, 0.323
D	Fear of childbirth	-0.040, 0.094	-0.030, 0.490	-0.011, 0.805	-0.010, 0.678	0.028, 0.502	0.049, 0.226	-0.034, 0.151	-0.072, 0.086	-0.059, 0.151
E	Previous caesarean delivery	0.014, 0.566	0.032, 0.711	0.060, 0.191	0.027, 0.251	-0.067, 0.119	-0.010, 0.803	0.032, 0.187	-0.008, 0.861	0.048, 0.253
R	Epidural anaesthesia at birth	-0.072, 0.089	-0.081, 0.069	-0.061, 0.183	**-0.114**,** 0.007**	**-0.106**,** 0.012**	-0.082, 0.055	**-0.099**,** 0.020**	**-0.092**,** 0.030**	-0.058, 0.180
M	Oxytocin use during labour	0.011, 0.659	0.022, 0.622	0.037, 0.419	-0.014, 0.559	-0.011, 0.789	-0.001, 0.975	-0.033, 0.173	-0.004, 0.927	0.006, 0.888
E	Episiotomy	-0.026, 0.279	-0.027, 0.531	-0.003, 0.936	**-0.093**,** < 0.001**	**-0.086**,** 0.035**	-0.055, 0.168	**-0.100**,** < 0.001**	**-0.108**,** 0.008**	**-0.083**,** 0.041**
D	Duration of labour	-0.024, 0.320	-0.017, 0.687	-0.026, 0.543	-0.001, 0.968	0.062, 0.125	¨0.058, 0.138	0.014, 0.550	0.040, 0.318	0.028, 0.485
I	Mode of delivery	**-0.130**,** < 0.001**	**-0.145**,** < 0.001**	**-0.111**,** 0.011**	**-0.284**,** < 0.001**	**-0.308**,** < 0.001**	**-0.258**,** < 0.001**	**-0.295**,** < 0.001**	**-0.297**,** < 0.001**	**-0.255**,** < 0.001**
A	Bleeding > 1000 ml	**-0.093**,** < 0.001**	**-0.099**,** 0.020**	**-0.097**,** 0.023**	**-0.115**,** < 0.001**	**-0.124**,** 0.002**	**-0.116**,** 0.003**	**-0.105**,** < 0.001**	**-0.149**,** < 0.001**	**-0.151**,** < 0.001**
T	Perineal injury	**-0.072**,** 0.003**	-0.075, 0.077	-0.060, 0.158	**-0.072**,** 0.002**	-0.030, 0.459	-0.009, 0.816	**-0.075**,** 0.002**	-0.056, 0.163	-0.040, 0.315
O	Apgar score < 4 at 5 min	-0.038, 0.114	-0.012, 0.789	-0.014, 0.752	**-0.058**,** 0.015**	-0.001, 0.986	-0.006, 0.889	**-0.062**,** 0.010**	-0.017, 0.686	-0.027, 0.507
R	Birth weight	0.011, 0.654	0.071, 0.98	0.018, 0.692	-0.025, 0.305	0.006, 0.886	-0.048, 0.240	-0.020, 0.233	0.008, 0.847	-0.031, 0.455

Analysis of the data showed that women were more satisfied when they were multiparous (*P* < 0.001), gave birth normal vaginally (*P* = 0.009), and experienced less bleeding during labour (*p* < 0.001). Predictors co-habitation with a partner and perineal injury also showed an association with satisfaction with care at birth. However, after adjusting for other confounders and mediators, no relationship was found (Table 3).

### Women’s childbirth experiences, evaluated at 8 weeks and 1 year postpartum

When the data were analysed to evaluate the relationships between the childbirth experience score at 8 weeks after birth and the predictors cited above, the women reported a worse childbirth experience if they gave birth following IOL (*P* < 0.001), were nulliparous (*P* < 0.001), had a instrumental/operative delivery (*P* < 0.001), and experienced more bleeding at birth (*P* < 0.001). Certain predictors, such as epidural anaesthesia, oxytocin use during birth, episiotomy, perineal injury, an Apgar score below 7 at 5 min, and cohabitation with a partner, can affect childbirth experiences at 8 weeks and one year when unadjusted. However, when adjusting for other confounders and mediators, these associations may be influenced or obscured (see Table 3).

One year after birth, like the experience at 8 weeks postpartum, women reported a more positive childbirth experience if they gave birth after SOL (*P* < 0.001), were multiparous (*P* < 0.001), had a normal vaginal delivery (*P* < 0.001), and experienced less bleeding at birth (*P* < 0.001).

### Relationship between satisfaction with care at birth and childbirth experiences

We also investigated whether there was any relationship between satisfaction with care at birth, as reported at hospital discharge, and childbirth experiences at 8 weeks and 1 year postpartum. In both the IOL and SOL groups, women with a high score for childbirth experience already had a higher satisfaction score at birth. We found that the satisfaction score at birth can positively influence childbirth experiences at both 8 weeks (IOL: *P* < 0.001, SOL: *P* < 0.001) and 1 year postpartum (IOL: *P* < 0.001, SOL: *P* < 0.001). (Table 3)

## Discussion

In this retrospective registry-based study, we examined whether the induction of labour in late-term pregnant women reduces their satisfaction with care during birth and results in a less-positive childbirth experience than women in the SOL group. Our results indicated that patients in the IOL and SOL groups had different satisfaction scores with care at birth, showing a relationship between the method of labour commencement and satisfaction scores at hospital discharge. Women in the SOL group were more satisfied with care at birth and reported a more positive birth experience at both 8 weeks and 1 year after delivery compared to those in the IOL group. Furthermore, women’s childbirth experience scores in both groups were less positive 1 year after birth compared to 8 weeks postpartum.

Our findings suggest that a range of factors may predict women’s satisfaction with care at birth and their childbirth experience. These factors include the mother’s characteristics and the foetal-maternal health outcomes post-delivery. However, the factors associated with satisfaction and the childbirth experience varied at the three evaluation time points. Notably, multiparity, experience of less bleeding at birth, and non-instrumental vaginal delivery were consistent factors that increased VAS scores on all three occasions. A significant proportion of women did not answer the question about their childbirth experience at 8 weeks and/or 1 year after delivery (73% and 72%, respectively) and were excluded from the study. Excluding these women could lead to biased results, as there was a systematic difference between responders and non-responders (e.g., a higher proportion of multiparous women, women with previous cesarean sections, women born abroad, etc.). This is important to consider when interpreting the conclusions of the study, as if non-response is related to key factors, the results may not accurately reflect the experiences of the broader population.

Women’s childbirth experience is a complex process reflecting aspects of the quality of care she receives during labour and birth, based on the respect shown by healthcare providers, effective communication, and emotional support [26, 27]. Other factors, including the characteristics of the patient, can also determine the patient’s experience by affecting her needs, expectations, and values [28]. Another study suggested that even when a delivery starts spontaneously, the total birth experience can become negative if complications occur, irrespective of the particular complication; e.g., instrumental extraction, other operative requirement, heavy bleeding after delivery, or infection [7]. Many risk factors for a negative birth experience appear to be related to unexpected medical events and social background [29]. In our study, there were no significant differences between the IOL and SOL groups in the duration of the active phase of labour or in foetal and maternal complications. Since patients in both groups primarily consisted of healthy, low-risk women, the findings may have been influenced by the study population, limiting their generalizability to higher-risk populations. The absence of differences between the groups in the mentioned parameters likely reflects the the inherent characteristics of low-risk women rather than the mode of labour onset itself being the determining factor. However, in the SOL group, the rates of episiotomy, epidural anesthesia, and oxytocin use during birth were lower compared to the IOL group. Consequently, the birth experience differed between the groups and women in the SOL group reported a more positive birth experience than those in the IOL group.

Another interesting finding from our study is that the perceived birth experience in both the IOL and SOL groups worsened over time, being less positive at 1 year compared to 8 weeks post-delivery. In line with our results, previous studies have indicated that women who underwent IOL were generally less satisfied with their labour and had a more negative birth experience than those whose labour started spontaneously [14, 30], and many women associate IOL with feelings of anxiety, pain, discomfort, and a feeling of powerlessness [31, 32]. This group of women tends to be more anxious and less satisfied with their birth experience compared to those who experience spontaneous onset of labour [33, 34]. Our study supports these findings, revealing that women who underwent IOL reported a more negative childbirth experience compared to those in the SOL group.

A patient’s satisfaction at birth reflects their evaluation of the care they received during labour relative to their expectations, and childbirth experience reflects their interactions with caregivers and the healthcare system, and includes all events and conditions associated with their birth process [13]. Most previous studies did not distinguish between the maternal satisfaction with care and childbirth experience, so description of those is usually overlapping and not clearly delineated. Patient satisfaction with birth is a representative measure of a patient’s experience of care, health outcomes, and trust in the health system, and reflects whether the care received has met the patient’s needs and expectations [26, 35]. Patient satisfaction with hospital care is significantly affected by her interactions with the care provider, including emotional support, the surrounding physical environment, communication skills, the clear explanation of sufficient information, technical skills of staff (e.g., clinical competence and use of hospital equipment), foetal outcomes (e.g., maternal and newborn health), socioeconomic status, and reproductive history [36]. An earlier study suggested that when labour is induced, the birth experience is affected mainly by the progress of labour, in which the delay between induction and delivery plays a significant role. The method of labour induction, more frequent vaginal examinations, parity and caesarean delivery are considered secondary factors that may affect a woman’s satisfaction with birth [37]. Another study linked immigration and fear of childbirth with an increased risk [38, 39]. Many women experience anxiety about childbirth, with some facing intense fear that impacts their well-being and perception of pregnancy. Some may even hesitate to become pregnant. Early in pregnancy, women can share their concerns with a midwife during routine check-ups at maternity care centers, where they have the opportunity for discussions and can arrange contact with specialized caregivers to help manage their fears. Our study found no differences in the proportion of women experiencing fear of childbirth between the SOL and IOL groups. Although the IOL and SOL groups reported diverse birth experiences, the impact of these factors showed no significant difference between the groups.

Previous studies have supported the notion that the more negative aspects of childbirth may take longer to manifest in women [40]. The “halo effect” at birth refers to the phenomenon where the positive emotions and euphoria experienced after giving birth to a healthy baby can influence a mother’s memory of the pain and challenges of childbirth. This effect can make the overall birth experience seem more positive in retrospect, even if it was quite painful at the time. This highlights the importance of not only considering immediate outcomes but also the long-term effects of childbirth experiences. The differences among the results may be attributable to differences in the times at which satisfaction and the birth experience were evaluated. Our results show that, for example, epidural anaesthesia during labour had no effect on satisfaction with care at birth but was associated with a higher likelihood of a negative birth experiences at 8 weeks and 1 year after delivery.

A key limitation of this study was the use of a single VAS question to assess patient satisfaction and birth experience. While this method is routinely employed by Swedish caregivers, it does not capture qualitative factors [14]. Additionally, as a registry-based study, missing data, variations in maternity-care routines, and the unavailability of certain information—such as intrapartum complications and labour duration—may introduce inconsistencies. Another constraint was the lack of access to data from the Swedish Neonatal Care Register (SNQ) on newborn transfers to the NICU, which limited the accurate assessment of NICU admissions. However, a preliminary analysis of newborn diagnosis codes for newborns requiring immediate NICU admission after birth in the pregnancy registry showed no differences between the groups. Nevertheless, some uncertainty remains, necessitating further research. Similarly, data on labour induction methods were unavailable, preventing evaluation of their impact. One study suggests that oral induction methods may yield higher satisfaction [30], however this study could not explore such differences. The study was partly conducted during the COVID-19 pandemic, when maternity care units restricted who could be present in the delivery room, potentially influencing childbirth experiences. Additionally, women with incomplete satisfaction or birth experience data were excluded, creating uncertainty about their experiences. Survey response rates were 56.6% at 8 weeks and 51.1% at 1 year postpartum [41], which may limit the generalizability of findings and introduce bias, affecting the accuracy of conclusions [42].

## Conclusion

The results of this study showed differences in satisfaction with care during labour between the SOL and IOL groups, suggesting a relationship between the method of labour commencement and satisfaction scores at the time of hospital discharge. Women in the SOL group were more satisfied with care at birth and reported a more positive birth experience at both 8 weeks and 1 year after delivery compared to those in the IOL group. Furthermore, women’s childbirth experience scores, regardless of the method of labour commencement, may change over time, becoming less positive 1 year after birth compared to 8 weeks postpartum. Parity, heavy bleeding at birth, and mode of delivery were the predictors showing associations with satisfaction with care at birth and childbirth experiences at 8 weeks and 1 year.

Women’s feelings and assessments of their birth experience are complex and influenced by several factors, which could vary in effect depending on the time of assessment. It is essential for healthcare providers to address these factors to optimize the childbirth experience for all women, regardless of the method of labour onset.

## Data Availability

No datasets were generated or analysed during the current study.

## Abbreviations

SOL: Spontaneous onset of labour
IOL: Induction of labour
SKR: Sveriges kommuner och regioner
VAS: Visual analogue score
IUGR: Intra-uterine growth retardation
BMI: Body mass index
OR: Odds ratio
SD: Standard deviation
CI: Confidence interval
EDA: Epidural anaesthesia
PH: potential hydrogen
Β: Beta - coefficient
